# Cardioversion strategy impacts rate control during recurrences in patients with paroxysmal atrial fibrillation: A subanalysis of the RACE 7 ACWAS trial

**DOI:** 10.1002/clc.24161

**Published:** 2023-10-23

**Authors:** Rachel M. J. van der Velden, Nikki A. H. A. Pluymaekers, Elton A. M. P. Dudink, Justin G. L. M. Luermans, Joan G. Meeder, Wilfred F. Heesen, Timo Lenderink, Jos W. M. G. Widdershoven, Jeroen J. J. Bucx, Michiel Rienstra, Otto Kamp, Jurren M. van Opstal, Charles J. H. J. Kirchhof, Vincent F. van Dijk, Henk P. Swart, Marco Alings, Isabelle C. Van Gelder, Harry J. G. M. Crijns, Dominik Linz

**Affiliations:** ^1^ Department of Cardiology Maastricht University Medical Centre and Cardiovascular Research Institute Maastricht Maastricht The Netherlands; ^2^ Department of Cardiology RadboudUMC Nijmegen The Netherlands; ^3^ Department of Cardiology VieCuri Medical Center Noord‐Limburg Venlo The Netherlands; ^4^ Department of Cardiology Zuyderland Medical Center Heerlen The Netherlands; ^5^ Department of Cardiology Elisabeth‐TweeSteden Hospital Tilburg The Netherlands; ^6^ Department of Cardiology Diakonessenhuis Utrecht Utrecht The Netherlands; ^7^ Department of Cardiology, University of Groningen University Medical Center Groningen Groningen The Netherlands; ^8^ Department of Cardiology, Amsterdam Cardiovascular Sciences, Amsterdam UMC Vrije Universiteit Amsterdam The Netherlands; ^9^ Department of Cardiology Medical Spectrum Twente Enschede The Netherlands; ^10^ Department of Cardiology Alrijne Hospital Leiderdorp The Netherlands; ^11^ Department of Cardiology St. Antonius Hospital Nieuwegein The Netherlands; ^12^ Department of Cardiology Antonius Hospital Sneek The Netherlands; ^13^ Department of Cardiology Amphia Hospital Breda The Netherlands; ^14^ Department of Cardiology, Center for Heart Rhythm Disorders University of Adelaide and Royal Adelaide Hospital Adelaide Australia; ^15^ Department of Biomedical Sciences, Faculty of Health and Medical Sciences University of Copenhagen Copenhagen Denmark

**Keywords:** acute management, atrial fibrillation, cardioversion, mobile health, rate control

## Abstract

**Background:**

In the Rate Control versus Electrical Cardioversion Trial 7–Acute Cardioversion versus Wait and See, patients with recent‐onset atrial fibrillation (AF) were randomized to either early or delayed cardioversion.

**Aim:**

This prespecified sub‐analysis aimed to evaluate heart rate during AF recurrences after an emergency department (ED) visit identified by an electrocardiogram (ECG)‐based handheld device.

**Methods:**

After the ED visit, included patients (*n* = 437) were asked to use an ECG‐based handheld device to monitor for recurrences during the 4‐week follow‐up period. 335 patients used the handheld device and were included in this analysis. Recordings from the device were collected and assessed for heart rhythm and rate. Optimal rate control was defined as a target resting heart rate of <110 beats per minute (bpm).

**Results:**

In 99 patients (29.6%, mean age 67 ± 10 years, 39.4% female, median 6 [3–12] AF recordings) a total of 314 AF recurrences (median 2 [1–3] per patient) were identified during follow‐up. The average median resting heart rate at recurrence was 100 ± 21 bpm in the delayed vs 112 ± 25 bpm in the early cardioversion group (*p* = .011). Optimal rate control was seen in 68.4% [21.3%–100%] and 33.3% [0%–77.5%] of recordings (*p* = .01), respectively. Randomization group [coefficient −12.09 (−20.55 to −3.63, *p* = .006) for delayed vs. early cardioversion] and heart rate on index ECG [coefficient 0.46 (0.29–0.63, *p* < .001) per bpm increase] were identified on multivariable analysis as factors associated with lower median heart rate during AF recurrences.

**Conclusion:**

A delayed cardioversion strategy translated into a favorable heart rate profile during AF recurrences.

## Graphical abstract

In the RACE 7 ACWAS trial, patients with recent‐onset atrial fibrillation (AF) were randomized to either early or delayed cardioversion. This prespecified sub‐analysis evaluating heart rate during AF recurrences after an emergency department visit showed that a delayed cardioversion strategy translated into a favorable heart rate profile during AF recurrences.

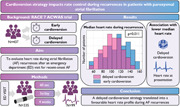

**Abbreviations**
AF: atrial fibrillation
ED: emergency department
RACE 7 ACWAS trial: Rate Control versus Electrical Cardioversion Trial 7–Acute Cardioversion versus Wait and See

## 1 INTRODUCTION

Atrial fibrillation (AF) may present with a variety of symptoms and can severely compromise daily functioning and quality of life,^1^, ^2^, ^3^, ^4^ often prompting patients to seek medical care. In the Rate Control versus Electrical Cardioversion Trial 7–Acute Cardioversion versus Wait and See (RACE 7 ACWAS),^5^ patients presenting with recent‐onset AF at the emergency department (ED) were randomized to either an early or a delayed cardioversion strategy. Within the delayed cardioversion strategy, more focus was given to initial rate control treatment at the ED. In general, rate control aims to alleviate symptoms and improve quality of life.^6^, ^7^ Adequate rate control remains advised in all AF patients, complementary to rhythm control strategies such as catheter ablation or antiarrhythmic drugs.^8^ Current European Society of Cardiology (ESC) guidelines for the management of AF advise a lenient rate control strategy as an acceptable initial approach with a target resting heart rate of <110 beats per minute (bpm) and more strict rate control if complaints persist.^6^, ^9^, ^10^ Whether a delayed cardioversion strategy also impacts the quality of rate control during recurrences of AF beyond the presenting episode, is currently unknown.

The aim of this prespecified sub‐analysis of the RACE 7 ACWAS trial^5^ was to evaluate heart rate during recurrences after an ED visit for recent‐onset AF using a mobile Health (mHealth) device and to assess factors associated with heart rate during recurrences.

## 2 METHODS

### 2.1 Study design

This study is a prespecified sub‐analysis of the RACE 7 ACWAS trial (NCT02248753). Briefly, the RACE 7 ACWAS trial was a multicentre, noninferiority randomized controlled trial comparing early to delayed cardioversion in patients with recent‐onset AF at the ED. Early cardioversion consisted of direct pharmacological or electrical cardioversion. Delayed cardioversion consisted of the administration of rate control medication to achieve symptom alleviation and a heart rate of <110 bpm. If spontaneous conversion did not occur, cardioversion was performed within 48 h from symptom onset. Detailed descriptions of the design of this study have been published elsewhere.^5^, ^11^ The RACE 7 ACWAS trial showed noninferiority of delayed cardioversion compared to early cardioversion in achieving a return to sinus rhythm at 4 weeks. The trial was approved by the ethical review board MUMC+/Maastricht University [NL number: 47065.068.13, METC number: 14‐2‐018] and complied with the Declaration of Helsinki. All participants provided written informed consent.

Patients in this trial were given an electrocardiogram (ECG)‐based handheld device (MyDiagnostick, Applied Biomedical Systems) to monitor for recurrences during the 4‐week follow‐up period. Patients were instructed on how to use the device and were asked to record 1 min heart rate and rhythm recordings three times a day and in case of symptoms. The overall adherence and adherence consistency of patients have been described previously.^12^ During the follow‐up, the recordings were stored in the device and after the 4‐week follow‐up period, all recordings were collected simultaneously. All recordings were reviewed by two independent researchers (R.V. and N.P.) for the presence of AF. In case of discrepancies, a third researcher (D.L.) was involved for the final decision. An episode of AF was registered as a recurrence if the duration of the episode was at least 30 s. Data from the mHealth device was censored at exactly 4 weeks.

### 2.2 Participants of the RACE 7 ACWAS trial

Participants of the RACE 7 ACWAS trial were adult (>18 years old) patients who presented to the ED with recent‐onset symptomatic AF episodes lasting <36 h. Patients were excluded in case of signs of myocardial infarction or heart failure, hemodynamic instability, and a history of either sick sinus syndrome, Wolff‐Parkinson‐White syndrome, persistent AF or unexplained syncope.

### 2.3 Procedures around rate control medication and cardioversion

Rate control strategy was described in the study protocol. For patients in the early cardioversion group, no specific instructions on the administration of rate control medication were given. First choice of treatment for early cardioversion was immediate pharmacological cardioversion. This was preferably performed with flecainide with an infusion scheme of 2 mg/kg body weight (150 mg maximum) in 10 min or until conversion to sinus rhythm occurred. Electrical cardioversion was performed in patients with contraindications to pharmacological cardioversion, as well as in patients with previous or current unsuccessful pharmacological cardioversion. For electrical cardioversion, a maximum of three shocks was performed from a biphasic defibrillator in synchronized mode, with energy set at 200, 360, and 360 J.

In the delayed cardioversion group, acute rate control was achieved through the administration of beta blockers (1st choice), calcium channel blockers (2nd choice) or digoxin (3rd choice). The dosage of the rate control medication was titrated based on heart rate, with the goal to achieve a heart rate of <110 bpm. The oral maintenance dose was subsequently determined based on the initial dosage needed to achieve adequate rate control.

All data on rate control medication use and adaptation were collected from the electronic patient record. During the index ED visit, medication use at that time was verified and recorded, as were any changes made during this visit. The same applied to the second‐day visit in the delayed cardioversion group. Rate control medication used at index visit included all medication with a start date before the index visit. To determine which rate control medication was used at the start of the follow‐up period, this information was complemented with details on newly started medication, stopped medication or changes in dosages during the index visit in case of early cardioversion, and the index visit and/or the second‐day visit in case of delayed cardioversion.

### 2.4 Rate control assessment

For the assessment of rate control adequacy, current cut‐off values from the ESC guidelines were used, i.e. optimal rate control during the initial AF episode and subsequent AF recurrences was defined as a target resting heart rate of <110 bpm. For the calculation of the median heart rate during AF recurrences for a specific patient, all heart rates of all recordings during AF recurrences from that patient were used.

### 2.5 Statistical analysis

All continuous variables were tested for normality. Normally distributed variables are presented as mean ± standard deviation and were compared using a *t*‐test for independent samples, whereas nonnormally distributed variables are presented as median [interquartile range] and were compared using the Mann–Whitney *U* test. Categorical variables are presented as numbers (no.) with percentages (%) and were compared using a Chi‐square test or a Fisher's exact test, as appropriate. To determine factors associated with median heart rate during recurrences, unadjusted multivariable linear regression analysis was performed, including all variables that were significant in univariable analysis (*p* = .05) and those which were considered clinically relevant (i.e., rate control adaptation during index visit). A two‐sided *p* < .05 was considered statistically significant. All analyses were conducted using IBM SPSS Statistics, version 28 and MATLAB r2019b (The MathWorks).

## 3 RESULTS

Of the 437 patients (mean age 65 ± 11 years, 40.3% female) included in the RACE 7 ACWAS trial, 335 patients (mean age 65 ± 11 years, 41.8% female) used the ECG‐based handheld mHealth device. The remaining patients did not use the device due to unavailability at the time of inclusion. The mHealth monitoring identified 99 patients (29.6%, mean age 67 ± 10 years, 39.4% female) with at least one AF recurrence in the first 4 weeks, who were included in the current analysis. Baseline characteristics of these patients are presented in Table 1. In total, these patients had 314 recurrences (median number of recurrences 2 [1–3]) over 4 weeks follow‐up. Patients in the delayed cardioversion group more often had spontaneous conversion during the first 48 h after inclusion, which is inherent to the delayed cardioversion treatment strategy (73.5% vs. 12.0%, *p* < .001) and less recurrences during follow‐up (median 1 [1, 2] vs. 2 [1–5], *p* = .020). Of the other 56 patients, in 29 patients pharmacological cardioversion was attempted for their AF episode in the RACE 7 ACWAS trial (28 in early cardioversion group, 1 in delayed cardioversion group). This was successful in 22 patients, whereas 8 patients in the early cardioversion group underwent additional electrical cardioversion. A total of 34 patients underwent electrical cardioversion (22 in the early cardioversion group, 12 in the delayed cardioversion group) of which 1 in the early cardioversion group was unsuccessful.

**Table 1:** Characteristics of patients per randomization group.

	Total (*n* = 99)	Delayed cardioversion (*n* = 49)	Early cardioversion (*n* = 50)	*p* value
**Demographic factors and medical history at index visit**				
Age in years	67 ± 10	67 ± 8	67 ± 11	0.638
Female	39 (39.4)	20 (40.8)	19 (38.0)	0.774
BMI in kg/m^2^	26.5 [24.0–29.4]	26.7 [23.7–29.3]	26.3 [24.1–29.7]	0.771
Hypertension	53 (53.5)	22 (44.9)	31 (62.0)	0.088
Diabetes	14 (14.1)	6 (12.2)	8 (16.0)	0.592
Coronary artery disease	20 (20.2)	11 (22.4)	9 (18.0)	0.581
Heart failure	2 (2.0); *n* = *98*	2 (4.1)	0; *n* = *49*	0.495
Chronic obstructive pulmonary disease	7 (7.1)	4 (8.2)	3 (6.0)	0.715
Stroke or TIA	5 (5.1)	2 (4.1)	3 (6.0)	1.000
CHA_2_DS_2_‐VASc score	2 [1–3]	2 [1–3]	2 [1–4]	0.420
**AF characteristics at index visit**				
First detected AF at index visit	35 (35.4)	16 (32.7)	19 (38.0)	0.578
Previous electrical cardioversion	20 (22.2); *n* = *90*	11 (23.9); *n* = *46*	9 (20.5); *n* = *44*	0.693
Previous pharmacological cardioversion	22 (22.2)	8 (16.3)	14 (28.0)	0.162
Previous ablation	17 (17.2)	9 (18.4)	8 (16.0)	0.755
**Follow‐up**				
Spontaneous conversion (within initial treatment strategy/first 48 h)	43 (43.4)	36 (73.5)	7 (14.0)	**<0.001**
Number of AF recordings on handheld device per patient	6 [3–12]	5 [2–16]	6 [3–11]	0.558
Number of recurrences per patient	2 [1–3]	1 [1, 2]	2 [1–5]	**0.020**
Heart rate on measurement preceding AF recurrence (bpm)	64 [58–70]; *n* = *97*	63 [58–73]; *n* = *47*	64 [58–69]	0.882
Patients with an ED visit due to an AF recurrence within the first 4 weeks	20 (20.2)	9 (18.4)	11 (22.0)	0.653
Patients with cardiovascular or cerebral complications^a^	5 (5.1)	4 (8.2)	1 (2.0)	0.204

*Note*: Data in mean ± standard deviation, median [interquartile range] or numbers (%). The number in italics after the semicolon indicates the available data for that variable. Bold values indicate statistically significant at *p* < 0.05.

Abbreviations: AF, atrial fibrillation; BMI, body mass index; bpm, beats per minute; ECG, electrocardiogram; ED, emergency department; HR, heart rate; TIA, transient ischemic attack.

a One patient was admitted because of suspected acute coronary syndrome with negative analysis (D), one patient was admitted for observation of a broad complex tachycardia after flecainide administration (D), one patient was admitted for observation of a collapse before discharge (E), one patient was admitted for colonoscopy because of rectal bleeding after initiation of anticoagulation (D) and one patient had a sinus arrest after flecainide infusion for an AF recurrence for which 30 seconds of compressions were performed, after which cardiac output was restored (D). Abbreviations: D = delayed cardioversion group, E = early cardioversion group.

Patients with a median heart rate <110 bpm during AF recurrences had lower heart rates on the index ECG (106 [95–127] vs. 137 [119–152], *p* = <.001), had more often undergone a previous electrical cardioversion (30.6% vs. 12.5%, *p* = .042) and were more often in the delayed cardioversion group (61.5% vs. 37.0%, *p* = .015). There were no significant differences in rate control medication use during the index visit or at the beginning of the follow‐up period between the two groups (Table S1). Detailed information on rate control medication use and adaptation is provided in Table S2.

### 3.1 Assessment of heart rate during AF recurrences by randomization group

**Figure 1:**
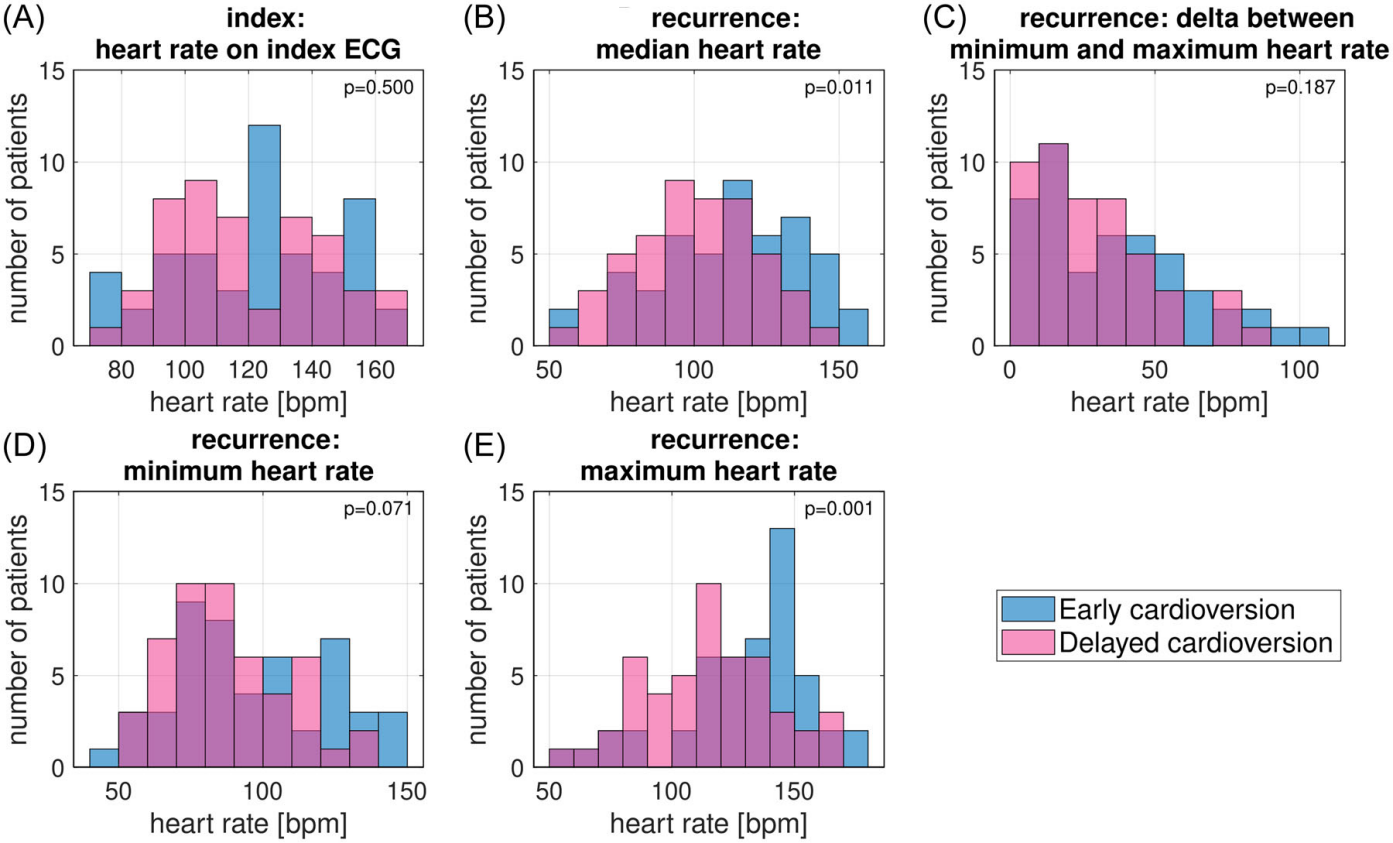
Heart rate on index ECG, minimum, median, maximum and delta heart rate during recurrences by randomization group (early vs. delayed cardioversion). (A–E) show histograms for heart rate characteristics for patients in the delayed (pink) and early (blue) cardioversion group. Purple bars indicate overlap between the two. bpm, beats per minute; ECG, electrocardiogram.

Figure 1 shows details on rate control during AF recurrences per patient, including minimum, maximum and median heart rate as well as the delta between the minimum and maximum heart rate for all AF recordings per patient combined. Mean heart rate on index ECG and the number of patients with heart rate <110 bpm on index ECG were not statistical significantly different between the two randomization groups. In addition, there were no statistically significant differences in use or adaptation of medication. However, the average median heart rate during recurrences was significantly lower in patients in the delayed cardioversion group compared to the early cardioversion group (100 ± 21 vs. 112 ± 25, *p* = .011) and in accordance, there were significantly more patients with median heart rate <110 bpm in the delayed cardioversion group (65.3% vs. 40.0%, *p* = .015) (Table 2). Additionally, the maximum heart rate was also significantly lower in the delayed cardioversion group (115 [94–131] vs. 136 [118–147], *p* = .001). Neither the number of patients on rate control medication during index visit or at the start of the follow‐up period, nor the median dosage of rate control medication differed significantly between the two randomization groups. Detailed information on medication adaptation is provided in Table S3. Detailed information on medication dosage of beta blockers and sotalol (normalized to metoprolol equivalent doses) is provided in Figure S1.

**Table 2:** Heart rate on index electrocardiogram and during AF recurrences and medication use and adaptation during the index visit by randomized group.

	Total (*n* = 99)	Delayed cardioversion (*n* = 49)	Early cardioversion (*n* = 50)	*p* value
**Index visit**				
Heart rate on index ECG in bpm	121 ± 25	119 ± 24	122 ± 25	0.500
Patients with heart rate <110 bpm on index ECG	37 (37.4)	21 (42.9)	16 (32.0)	0.264
Medication use	Rate control^a^: 55 (55.6)	Rate control: 29 (59.2)	Rate control: 26 (52.0)	0.472
	BB: 37 (37.4)	BB: 21 (42.9)	BB: 16 (32.0)	0.264
	Digoxin: 2 (2.0)	Digoxin: 0	Digoxin: 2 (4.0)	0.495
	NCCB: 4 (4.0)	NCCB: 1 (2.0)	NCCB: 3 (6.0)	0.617
	AAD class 3:13 (13.1)	AAD class 3:7 (14.3)	AAD class 3:6 (12.0)	0.774
Medication dosage in mg^b^	BB^c^: 62.5 [50–100]	BB^c^: 75 [50–100]	BB^c^: 62.5 [50–100]	0.765
	Sotalol: 100 [80–160]	Sotalol: 140 [70–180]	Sotalol: 80 [80–180]	0.699
Rate control medication adapted	25 (25.3)	15 (30.6)	10 (20.0)	0.224
**Start follow‐up period**				
Medication use	Rate control: 70 (70.7)	Rate control: 37 (75.5)	Rate control: 33 (66.0)	0.299
	BB: 44 (44.4)	BB: 24 (49.0)	BB: 20 (40.0)	0.369
	Digoxin: 3 (3.0)	Digoxin: 1 (2.0)	Digoxin: 2 (4.0)	1.000
	NCCB: 6 (6.1)	NCCB: 3 (6.1)	NCCB: 3 (6.0)	1.000
	AAD class 3:19 (19.2)	AAD class 3:10 (20.4)	AAD class 3:9 (18.0)	0.761
Medication dosage in mg^b^	BB^c^: 50 [50–100]	BB^c^: 100 [50–100]	BB^c^: 50 [50–94]	0.076
	Sotalol: 100 [80–160]	Sotalol: 120 [80–160]	Sotalol: 80 [80–160]	1.000
**During AF recurrence**				
Median heart rate in bpm	106 ± 24; *n* = *98*	100 ± 21	112 ± 25; *n* = *49*	**0.011**
Patients with median heart rate <110 bpm	52 (52.5); *n* = *98*	32 (65.3)	20 (40.8); *n* = *49*	**0.015**
Maximum heart rate in bpm	125 [107–144]; *n* = *98*	115 [94–131]	136 [118–147]; *n* = *49*	**0.001**
Minimum heart rate in bpm	91 ± 24; *n* = *98*	87 ± 21	95 ± 27; *n* = *49*	0.071
Delta between minimum and maximum heart rate in bpm	27 [14–45]; *n* = *98*	24 [12–40]	33 [15–53]; *n* = *49*	0.187

*Note*: Data in mean ± standard deviation, median [interquartile range] or numbers (%). The number in italics after the semicolon indicates the available data for that variable. Two recurrences from one patient were excluded because heart rate during recurrences could not be adequately assessed. Bold values indicate statistically significant at *p* < 0.05.

Abbreviations: AAD, antiarrhythmic drugs; BB, beta blocker; bpm, beats per minute; ECG, electrocardiogram; NCCB, non dihydropyridine calcium channel blocker.

^a^ Rate control medication = BB, digoxin, NCCB (verapamil) or class III AADs (amiodarone or sotalol).

^b^ Only calculated for BB and sotalol (1 patient using amiodarone was excluded). Median dosages of digoxin and NCCB were not calculated due to small numbers (since BB adaptation was defined as first choice in the protocol).

^c^ Normalized to metoprolol equivalent doses. At index 27 patients used metoprolol, 6 used bisoprolol, 2 used atenolol and 2 used propranolol. If a beta blocker was newly started this was always metoprolol, except in one patient (bisoprolol).

### 3.2 Assessment of optimal rate control

**Figure 2:**
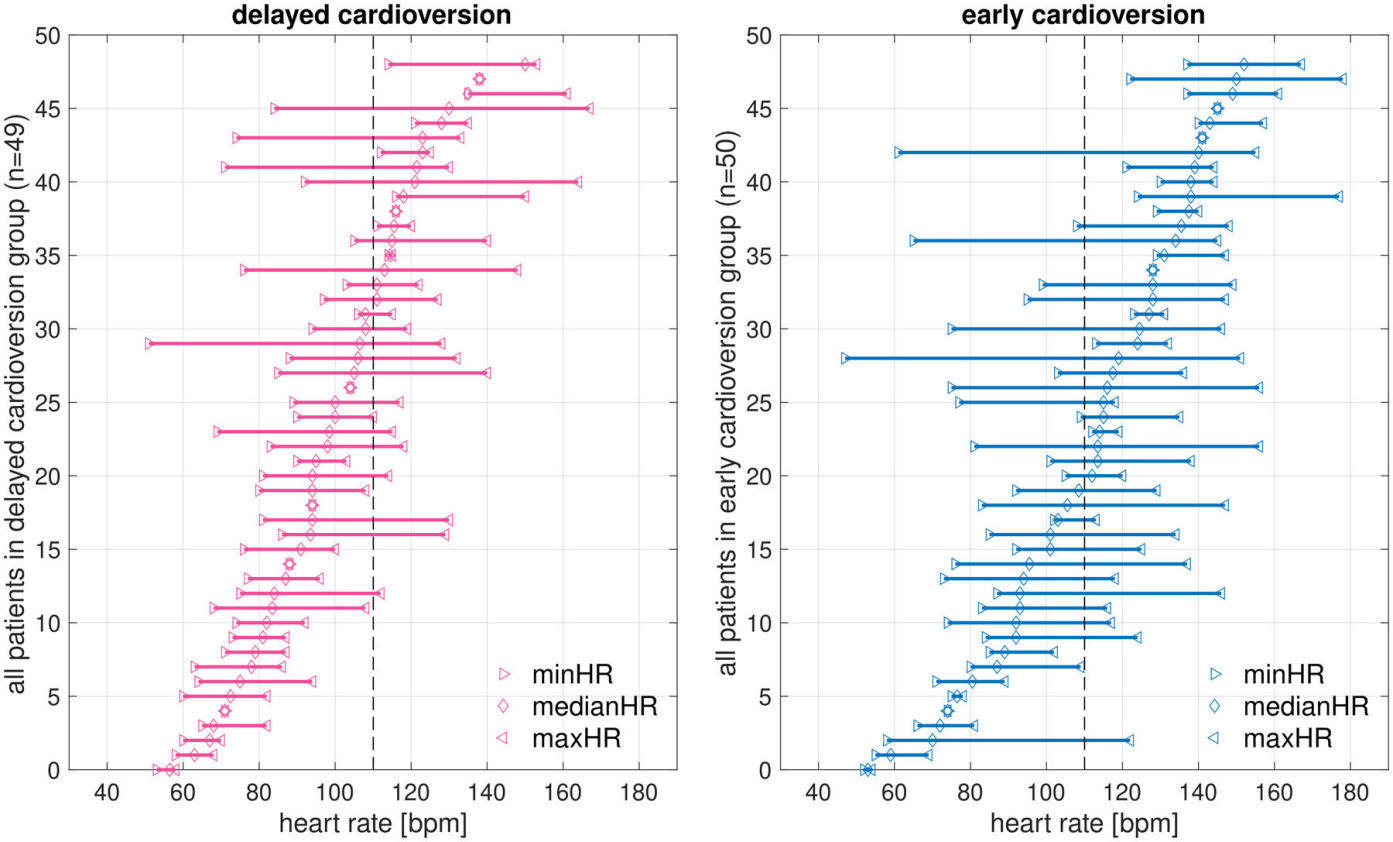
Heart rate profiles during recurrence of all patients individually. Figure 2 shows the minimum, median, and maximum heart rate, as well as the range between them, of all patients individually. A division line (dotted line) is set at 110 bpm. Patients in whom the bar lies completely to the left side of the dotted line have optimal rate control during all recordings. Patients in whom the bar lies completely to the right side of the dotted line have nonoptimal rate control during all recordings. Patients in whom the bar crosses the line have varying rate control. The left panel shows all the individual patients in the delayed cardioversion group, whereas the right panel shows the individual patients in the early cardioversion group. bpm, beats per minute; maxHR, maximum heart rate; medianHR, median heart rate; minHR, minimum heart rate.

Figure 2 shows heart rate profiles during AF recurrences for all individual 99 patients. Overall, patients had optimal rate control (rate <110 bpm) in a median of 52.1 [7.5–100]% of recordings (per patient, median number of recordings: 6 [3–12]). With a median number of 5 [2–16] recordings in the delayed cardioversion group and 6 [3–11] in the early cardioversion group, optimal rate control was present in a median of 68.4 [21.3–100]% and 33.3 [0–77.5]% of recordings, respectively (*p* = .01). Rate control was optimal in 100% of the recordings in 26 patients (26.5%), of whom 18 were in the delayed and 8 in the early cardioversion group. Rate control was never optimal in 20 patients (20.4%), of whom 7 were in the delayed and 13 were in the early cardioversion group (*p* = .021).

### 3.3 Factors associated with median heart rate during recurrences on multivariable linear regression analysis

Randomization group and heart rate on index ECG were identified on multivariable analysis as factors associated with median heart rate during recurrences. The median heart rate during recurrences increased with 0.46 (95% confidence interval: 0.29–0.63) bpm for every bpm increase in heart rate on the index ECG (*p* < .001) and was 12.09 (95% confidence interval: 3.63–20.55) bpm lower if patients were in the delayed cardioversion group compared to the early cardioversion group (*p* = .006). Outcomes of multivariable linear regression with respect to variables associated with the percentage of recordings with optimal rate control were similar to those described in Table 3.

**Table 3:** Univariable and multivariable factors associated with median heart rate during recurrences.

	Univariable		Multivariable	
	Coefficients (95% CI)	*p* value	Coefficients (95% CI)	*p* value
Delayed cardioversion group	−12.22 (−21.53 to −2.92)	**.011**	−12.09 (−20.55 to −3.63)	**.006**
Previous electrical cardioversion	−12.59 (−24.49 to −0.69)	**.038**	−7.99 (−18.11 to 2.14)	.120
Heart rate on index ECG	0.47 (0.30 to 0.64)	**<.001**	0.46 (0.29 to 0.63)	**<.001**
Rate control adaptation during index visit	−1.00 (−12.05 to 10.04)	.858	−4.82 (−14.54 to 4.91)	.327

*Note*: Bold values indicate statistically significant at *p* < 0.05.

Abbreviations: CI, confidence interval; ECG, electrocardiogram.

## 4 DISCUSSION

In this study, in patients with recent‐onset AF presenting at the ED, a delayed cardioversion strategy translated into a more optimal rate control during AF recurrences compared to early cardioversion without significant group‐differences in baseline or adjusted use of rate control medication. With a delayed cardioversion strategy, median heart rate at AF recurrence was lower as were peak heart rates. Apart from the randomized treatment, heart rate at presentation was independently associated with heart rate during AF recurrences.

Aiming for optimal rate control in AF patients to achieve symptom alleviation remains a main pillar of AF treatment strategies.^6^ Complementary to rhythm control strategies with catheter ablation or antiarrhythmic drugs, the RACE 7 ACWAS findings support acute management at the ED. Data on spontaneous conversion can help to determine the degree of the underlying AF substrate and may provide interesting information to help decide on the best rhythm control strategy. The data provided in this prespecified sub‐analysis of the RACE 7 ACWAS trial provides information about feasibility and effectivity of rate control within the early and delayed cardioversion strategy.

First, a lower heart rate at the index visit was independently associated with lower median heart rate during recurrences in both randomization arms. Interestingly, these patients with lower heart rate during the index visit did not use more rate control medication compared to those with higher heart rates at the index visit. Although beta blocker dosage was numerically higher in those with optimal heart rate at index, the proportion of patients using beta blocker medication was significantly larger in those with nonoptimal heart rates. This suggests that the heart rate during AF recurrences reflects the typical heart rate during AF for a given patient rather than the effect of rate control medication alone.

As one of the main findings of this manuscript, we describe significantly lower maximum heart rates during recurrences in the delayed cardioversion group. Although we did not find any statistically significant differences in rate control medication use, dosage or adaptation between the early and delayed cardioversion group, numerically more patients in the delayed cardioversion group received rate control drugs at higher dosage or had their rate control medication adjusted. Indeed, in the delayed cardioversion group there was more focus on titrating rate control medication in the initial phase already. Although on a group level significant differences are absent, a combination of these factors may make a difference in the individual patient. Moreover, a potential power problem due to the small sample sizes should be kept in mind. Nonetheless, our study showed that titration of rate control medication is still suboptimal in many patients, and a more personalized titration of rate control medication based on heart rate, possibly guided by mHealth supported remote heart rate and rhythm monitoring, could achieve better rate control in all patients.

In addition to a combination of (1) a numerically higher number of patients with lower heart rates at index visit and (2) numerically more index use and adaptation of rate control medication, also the development of better coping mechanisms may have determined rate during AF recurrences in the delayed cardioversion group. Whilst both groups have had reassuring information about the often transient and self‐terminating nature of recent‐onset AF, patients in the delayed cardioversion group have been exposed to AF‐related symptoms for a longer time initially, which allows them to get to learn their AF and even experience spontaneous conversion of AF, which helps them to develop possible coping mechanisms. This may result in less stress and consequently in lower heart rates during AF compared to those in the early cardioversion group.^13^ On the other hand, lower heart rates during AF recurrences may also result in less symptoms during AF,^14^, ^15^, ^16^ which may reduce stress throughout the AF episode. Indeed, previous studies showed that in educated patients with increased self‐care and coping, a reduction in anxiety could be observed^17^ and symptom‐induced distress reducing techniques frequently led to a reduction in heart rate.^18^, ^19^, ^20^ Although we did not perform any specific questionnaires to objectively assess stress during the 4‐week study period, above mentioned psycho‐behavioral factors may contribute to the association between delayed cardioversion and lower median heart rate during recurrences.

Irrespective of the involved mechanisms, a delayed cardioversion strategy may represent a useful and effective approach to optimize rate control in recent‐onset AF patients. To further harmonize the optimized rate control in the delayed cardioversion group, the mHealth approach used in this study could be transferred from an observational to an active telemonitoring system to allow early identification of actionable data (too slow or too fast heart rate during AF recurrence) and integration of this data into clinical decision pathways enabling faster responses and real time guidance of rate and rhythm control medication adaptation.^21^, ^22^ In a recent analysis we demonstrated, that patients in this clinical scenario show good adherence and adherence consistency, with a median adherence rate of 83.3% and the majority of patients performing measurements over the entire monitoring period.^12^ Remote rate control assessment through mHealth may increase patient involvement, may reduce stress and anxiety and may, if carefully instructed, even promote self‐management and self‐treatment.^23^ However, further studies are needed to define the best strategy how to integrate such a remote mHealth pathway and to test whether future ED visits can be prevented.

## 5 LIMITATIONS

In this study, heart rate and rhythm recordings were collected for 4 weeks by the patients via intermittent monitoring using an ECG‐based handheld mHealth device. Although beneficial in terms of accessibility and patient convenience, it does not supply continuous data, and the best time points and frequency of recordings to optimally use such a monitoring approach in this specific clinical scenario remains unclear. In addition, the short‐term follow‐up period of 4 weeks only limits transferability to long‐term results. We did not apply any anxiety‐specific questionnaires and can therefore not confirm whether the positive effect of a delayed cardioversion approach can indeed partly be attributed to reduced anxiety and the development of better coping mechanisms in these patients. Drug titration was found to be suboptimal in many cases, given the fact that rate control therapy was (at least partly) inadequate in 73.5% of patients. Irrespective of randomization group, more adequate drug titration should be pursued. Lastly, some subgroup analysis should be interpreted with caution, given the sample size.

## 6 CONCLUSION

A delayed cardioversion strategy translated into a more favorable heart rate profile during AF recurrences in patients with symptomatic recent‐onset AF.

## CONFLICT OF INTEREST STATEMENT

JL has a consultancy agreement with Medtronic and has received speakers fee from Bayer and Novartis, and a research grant ZonMW LEAP trial project number 852002101. MR has received consultancy fees from Medtronic, Arca Biopharma Inc, and Roche to the institution; MR has received unrestricted research grant from ZonMW and the Dutch Heart Foundation; DECISION project 848090001; from the Netherlands Cardiovascular Research Initiative: an initiative with support of the Dutch Heart Foundation; RACE V (CVON 2014–9), RED‐CVD (CVON2017‐11); from Top Sector Life Sciences & Health to the Dutch Heart Foundation (PPP Allowance; CVON‐AI (2018B017)); from the European Union's Horizon 2020 research and innovation program under grant agreement: EHRA‐PATHS (945260). IVG has received consultancy fees Institute from Medtronic, Daiichi, BMS, Bayer and Roche to the institution. IVG has received unrestricted research grants from the Netherlands Cardiovascular Research Initiative: an initiative with support of the Dutch Heart Foundation; from the European Union's Horizon 2020 research and innovation program under grant agreement: EHRA‐PATHS (945260). DL has received consultancy fees from Medtronic, Biotronik, Bayer and Itamar to the University of Maastricht. DL has received unrestricted research grant from the European Union's Horizon 2020 research and innovation program under grant agreement: EHRA‐PATHS (945260); from the Novo Nordisk Foundation; NNF Young Investigator Awards 2021 under grant agreement NNF21OC0066480. The other authors declare no conflicts of interest.

## Supporting information

Supporting information.

## Data Availability

Data are available on reasonable request. Data will be made available after an application at carim‐office@maastrichtuniversity.nl.
