# Supplementary material for: Cardioversion strategy impacts rate control during recurrences in patients with paroxysmal atrial fibrillation: A subanalysis of the RACE 7 ACWAS trial
Source: Clin Cardiol. 2023 Oct 23;47(1):e24161. doi: 10.1002/clc.24161 (PMC10766137; doi:10.1002/clc.24161)
Supplement: Supplementary file 1 — Supporting information. [file CLC-47-e24161-s001.docx]

**Supplementary Table 1. Characteristics of patients with median heart rate during recurrences ≥110 versus <110 beats per minute.**

|  | **Median HR ≥110 bpm (n=46)** | **Median HR <110 bpm (n=52)** | **p-value** |
| --- | --- | --- | --- |
| **Demographic factors and medical history at index visit** | | | |
| Age in years | 67±9 | 67±10 | 0.680 |
| Female | 21 (45.7) | 18 (34.6) | 0.265 |
| Delayed cardioversion group | 17 (37.0) | 32 (61.5) | **0.015** |
| BMI in kg/m^2^ | 26.6 [24.5-30.0] | 26.5 [23.4-29.1] | 0.276 |
| Hypertension | 25 (54.3) | 27 (51.9) | 0.810 |
| Diabetes | 8 (17.4) | 6 (11.5) | 0.409 |
| Coronary artery disease | 7 (15.2) | 13 (25.0) | 0.230 |
| Heart failure | 1 (2.2) | 1 (2.0); *n=51* | 1.000 |
| Chronic obstructive pulmonary disease | 5 (10.9) | 2 (3.8) | 0.248 |
| Stroke or TIA | 4 (8.7) | 1 (1.9) | 0.183 |
| CHA_2_DS_2_-VASc score | 2 [1-4] | 2 [1-3] | 0.589 |
| **AF characteristics and medication at index visit** | | | |
| First detected AF at index visit | 17 (37.0) | 18 (34.6) | 0.809 |
| Previous electrical cardioversion | 5 (12.5); *n=40* | 15 (30.6); *n=49* | **0.042** |
| Previous pharmacological cardioversion | 9 (19.6) | 12 (23.1) | 0.672 |
| Previous ablation | 5 (10.9) | 12 (23.1) | 0.111 |
| Spontaneous conversion | 17 (37.0) | 26 (50.0) | 0.194 |
| Heart rate on index ECG | 137 [119-152] | 106 [95-127] | **<0.001** |
| Medication use at index visit^+^ | Rate control - 27 (58.7)  BB - 20 (43.5)  Digoxin - 0  NCCB - 2 (4.3)  AAD class 3 - 5 (10.9) | Rate control - 28 (53.8)  BB - 17 (32.7)  Digoxin - 2 (3.8)  NCCB - 2 (3.8)  AAD class 3 - 8 (15.4) | 0.629  0.272  0.497  1.000  0.511 |
| Rate control medication adaptation^+^ | 10 (21.7) | 15 (28.8) | 0.421 |
| **Follow-up** |  |  |  |
| Number of AF recordings on handheld device | 6 [3-10] | 6 [3-16] | 0.491 |
| Number of recurrences | 2 [1-3] | 2 [1-5] | 0.580 |
| Heart rate on measurement preceding AF recurrence (bpm) | 65 [59-73] | 62 [57-68]; *n=50* | 0.103 |
| Patients with an ED visit due to an AF recurrence within the first 4 weeks | 10 (21.7) | 10 (19.2) | 0.758 |
| Patients with cardiovascular or cerebral complications* | 4 (8.7) | 3 (5.8) | 0.703 |

*Data in mean* ± *standard deviation, median [interquartile range] or numbers (%). The number in italics after the semicolon indicates the available data for that variable. One patient was excluded from comparison because heart rate during recurrences could not be adequately assessed ^+^Rate control medication = BB, digoxin, NCCB or class III AADs (amiodarone or sotalol), *Composed of admission for: 1) heart failure, 2) ischemic stroke or TIA, 3) unstable angina or acute coronary syndrome, 4) bradycardia or hypotension, or 5) tachycardia. Abbreviations: AAD = antiarrhythmic drugs, AF = atrial fibrillation, BB = beta blocker, BMI= body mass index, bpm = beats per minute, ECG = electrocardiogram, ED = emergency department, HR = heart rate, NCCB = non dihydropyridine calcium channel blocker, TIA = transient ischemic attack.*

**Supplementary Table 2. Medication details for patients with median heart rate ≥110 and <110 bpm**

|  | **Total (n=99)** | **Median HR ≥110 bpm (n=46)** | **Median HR <110 bpm (n=52)** |
| --- | --- | --- | --- |
| Number of rate control medications used at index | 0 medications - 44 (44.4)  1 medication - 54 (54.5)  2 medications - 1 (1.0) | 0 medications - 19 (41.3)  1 medication - 27 (58.7)  2 medications - 0 | 0 medications - 24 (46.2)  1 medication - 27 (51.9)  2 medications - 1 (1.9) |
| Medication dosage at index visit in mg^†^ | BB^ - 62.5 [50-100]  AAD class 3 - 100 [80-160] | BB^ - 50 [50-100]  AAD class 3 - 80 [80-140] | BB^ - 100 [50-100]  AAD class 3 - 140 [80-220] |
| Rate control medication adaptation (newly started or increased) | All - 25 (25.3)  BB - 16 (16.2)  Digoxin - 1 (1.0)  NCCB - 2 (2.0)  AAD class 3 - 6 (6.1) | All - 10 (21.7)  BB - 7 (15.2)  Digoxin - 0  NCCB - 1 (2.2)  AAD class 3 - 2 (4.3) | All - 15 (28.8)  BB - 9 (17.3)  Digoxin - 1 (1.9)  NCCB - 1 (1.9)  AAD class 3 - 4 (7.7) |
| Medication started in rate control naïve patients | BB - 13 (13.1)  Digoxin - 0  NCCB - 1 (1.0)  Amiodarone - 0  Sotalol - 2 (2.0) | BB - 5 (10.9)  Digoxin - 0  NCCB - 1 (2.2)  Amiodarone - 0  Sotalol - 0 | BB - 8 (15.4)  Digoxin - 0  NCCB - 0  Amiodarone - 0  Sotalol - 2 (3.8) |
| Number of patients in whom dosage of rate control medication was increased | BB - 3 (3.0)  Digoxin - 0  NCCB - 0  Amiodarone - 0  Sotalol - 0 | BB - 2 (4.3)  Digoxin - 0  NCCB - 0  Amiodarone - 0  Sotalol - 0 | BB - 1 (1.9)  Digoxin - 0  NCCB - 0  Amiodarone - 0  Sotalol - 0 |
| Number of patients in whom medication was changed | BB to digoxin - 1 (1.0)  BB to sotalol - 4 (4.0) | BB to digoxin - 0  BB to sotalol - 2 (4.3) | BB to digoxin - 1 (1.9)  BB to sotalol - 2 (3.8) |
| Number of patients in whom medication was added | Verapamil to sotalol - 1 (1.0) | Verapamil to sotalol - 0 | Verapamil to sotalol - 1 (1.9) |
| Overall dosage increase in mg (newly started or increased) | BB^ - 50 [50-100]  AAD class 3 - 120 [80-160] | BB^ - 50 [50-100]  AAD class 3 - 120 [80 - .] | BB^ - 50 [37.5-75]  AAD class 3 - 120 [80-160] |
| Number of rate control medications used at start follow-up period | 0 medications - 29 (29.3)  1 medication - 68 (68.7)  2 medications - 2 (2.0) | 0 medications - 14 (30.4)  1 medication - 32 (69.6)  2 medications - 0 | 0 medications - 14 (26.9)  1 medication - 36 (69.2)  2 medications - 2 (3.8) |
| Number of patients using rate control medication at start of follow-up period | Rate control - 70 (70.7)  BB - 44 (44.4)  Digoxin - 3 (3.0)  NCCB - 6 (6.1)  AAD class 3 - 19 (19.2) | Rate control - 32 (69.6)  BB - 22 (47.8)  Digoxin - 0  NCCB - 3 (6.5)  AAD class 3 - 7 (15.2) | Rate control - 38 (73.1)  BB - 22 (42.3)  Digoxin - 3 (5.8)  NCCB - 3 (5.8)  AAD class 3 - 12 (23.1) |
| Medication dosage at start of follow-up period in mg^†^ | BB^ - 87.5 [50-100]  AAD class 3 - 100 [80-160] | BB^ - 62.5 [50-100]  AAD class 3 - 80 [80-160] | BB^ - 100 [43.8-100]  AAD class 3 - 140 [80-160] |

*Data in median [interquartile range] or numbers (%). ^†^Only shown for beta blockers and sotalol (AAD class 3) because of the small sample sizes of the other medications. ^In metoprolol equivalent doses. Abbreviations: AAD = antiarrhythmic drugs, BB = beta blocker, NCCB = non dihydropyridine calcium channel blocker*

**Supplementary Table 3. Medication details for patients in the delayed and early cardioversion group**

|  | **Delayed cardioversion (n=49)** | **Early cardioversion (n=50)** |
| --- | --- | --- |
| Number of rate control medications used at index | 0 medications - 20 (40.8)  1 medication - 29 (59.2)  2 medications - 0 | 0 medications - 24 (48.0)  1 medication - 25 (50.0)  2 medications - 1 (2.0) |
| Rate control medication adaptation (newly started or increased) | All - 15 (30.6)  BB - 9 (18.4)  Digoxin - 1 (2.0)  NCCB - 2 (4.1)  AAD class 3 - 3 (6.1) | All - 10 (20.0)  BB - 7 (14.0)  Digoxin - 0  NCCB - 0  AAD class 3 - 3 (6.0) |
| Medication started in rate control naïve patients | BB - 6 (12.2)  Digoxin - 0  NCCB - 1 (2.0)  Amiodarone - 0  Sotalol - 1 (2.0) | BB - 7 (14.0)  Digoxin - 0  NCCB - 0  Amiodarone - 0  Sotalol - 1 (2.0) |
| Number of patients in whom dosage of rate control medication was increased | BB - 3 (6.1)  Digoxin - 0  NCCB - 0  Amiodarone - 0  Sotalol - 0 | BB - 0  Digoxin - 0  NCCB - 0  Amiodarone - 0  Sotalol - 0 |
| Number of patients in whom medication was changed | BB to digoxin - 1 (2.0)  BB to sotalol - 2 (4.1) | BB to digoxin - 0  BB to sotalol - 2 (4.0) |
| Number of patients in whom medication was added | Verapamil to sotalol - 1 (2.0) | Verapamil to sotalol - 0 |
| Overall dosage increase in mg (newly started or increased) *^†^* | BB^ - 50 [50-100]  AAD class 3 - 80 [80 - .] | BB^ - 50 [25-50]  AAD class 3 - 160 [80 - .] |
| Number of rate control medications used at start follow-up period | 0 medications - 12 (24.5)  1 medication - 36 (73.5)  2 medications - 1 (2.0) | 0 medications - 17 (34.0)  1 medication - 32 (64.0)  2 medications - 1 (2.0) |

*Data in median [interquartile range] or numbers (%). ^†^Only shown for beta blockers and sotalol (AAD class 3) because of the small sample sizes of the other medications. ^In metoprolol equivalent doses. Abbreviations: AAD = antiarrhythmic drugs, BB = beta blocker, NCCB = non dihydropyridine calcium channel blocker*


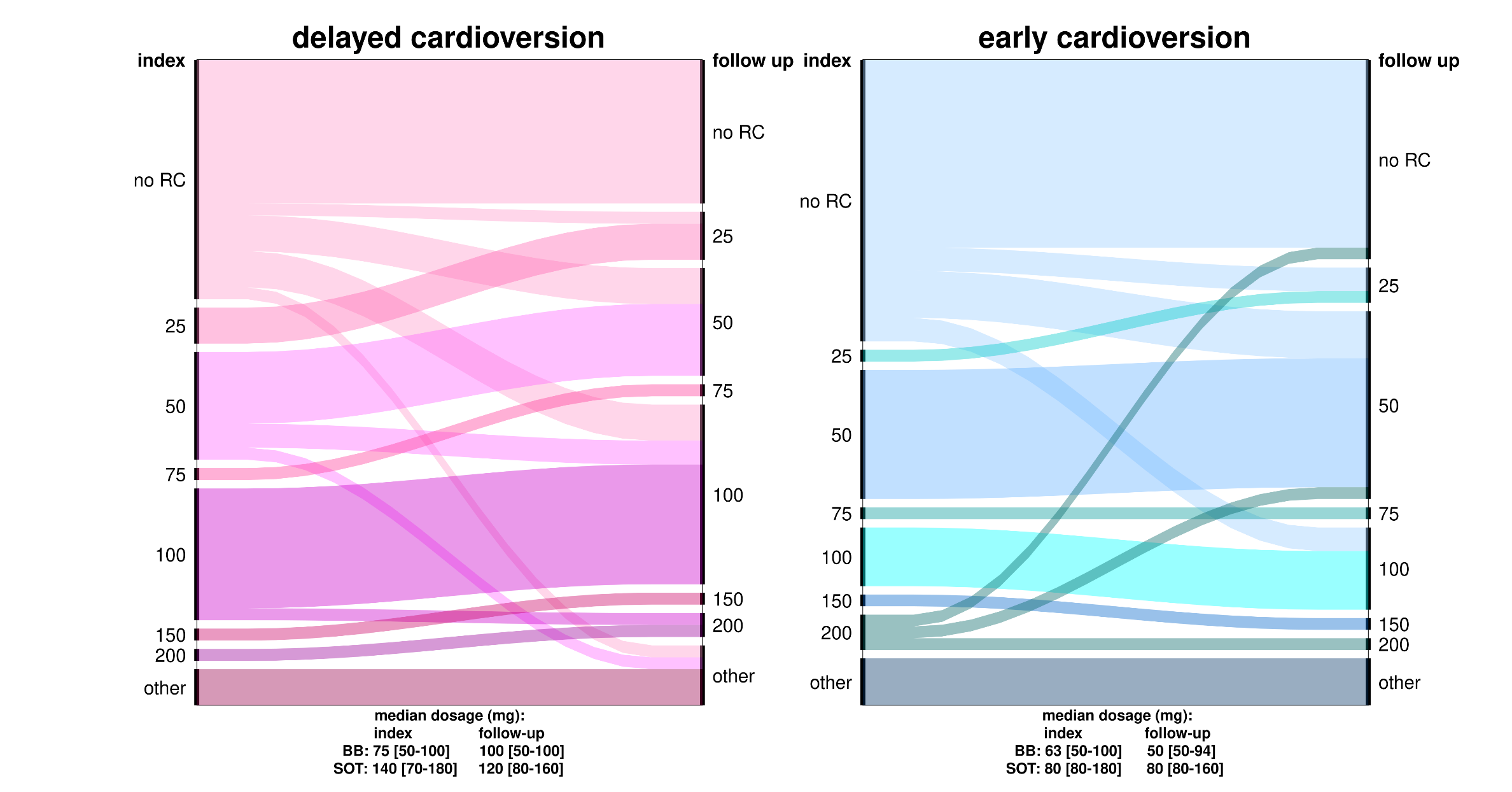
**Supplementary Figure 1. Sankey diagram summarizing the medication dosages for beta blockers and sotalol in metoprolol equivalent doses at index visit and at the start of the follow-up period for 49 delayed cardioversion and 50 early cardioversion patients**

Supplementary Figure 1 shows medication dosage in milligrams for beta blockers and sotalol in metoprolol equivalent doses at index (left side of figures) and at the start of the follow-up period (right side of figures). No RC = patients without any form of rate control medication. Other = patients on non-dihydropyridine calcium channel blockers, digoxin, amiodarone and one patient with unknown dosage of metoprolol. Abbreviations: BB = beta blocker, RC = rate control, SOT = sotalol
